# Hospital and Institutional News

**Published:** 1918-10-26

**Authors:** 


### HOSPITAL AND INSTITUTIONAL NEWS.

### THE KING'S SPEECH TO AMERICAN SOLDIERS.

The first Royal visit to a big American General Hospital was paid by the King and Queen on October 17 when they drove to the American Base Hospital No. 37, near Dartford. The Commandant, Colonel Fiske, explained that it had been formed in America with the original intention of being offered to the British Government, but that, on America's entry into the war, the hospital was transferred to the use of American soldiers. Colonel Fiske also said how much the Metropolitan Asylums Board had helped the institution, and publicly thanked the military authorities at Woolwich and the Joint Women's V.A.D. Detachment for their assistance. He added further that he and his staff "had received every imaginable kindness," and that only "the wonderful help and patience" of His Majesty's subjects had "made the apparently impossible possible to us." In reply the King said: "We in turn take off our hats to the great American nation which has rendered, is rendering, and is prepared to render such efficient and valuable aid to the common cause."

### THE THRONE AND THE PRESS.

How thankful we are that His Majesty represents his people and is the true spokesman of their feelings can be best shown by contrasting with the above speech a remark made in our hearing a few days ago. A man was passing by a house over which the Union Jack and the American Stars and Stripes were flying. The man said to his companion: "That house is inhabited by an American. These aliens ought not be allowed to remain in the country." In the speaker's hand was a copy of a daily newspaper which contained news of the American advance in France. To this abysmal fool, however, who was gloating over the splendid news, all the Allies of England are "aliens," and the fact that England and France owe their present superiority to the arrival of the American armies in Europe was nothing compared with the reckless rancour inculcated by the newspaper which he was reading in regard to aliens generally. The man who could utter such a statement is a public danger, and the type is being multiplied by the newspaper Trust which lives upon it. The worst of all Governments is newspaper government, but fortunately for Englishmen there towers above it the throne of King George.

### DEATH OF MR. HOWGRAVE GRAHAM.

The death of Mr. Howgrave Graham removes from the hospital world one of its oldest members. For five years from 1877 secretary to the Home Hospitals Association for Paying Patients, Mr. Graham became in 1882 secretary to the Hospital for Epilepsy and Paralysis, then at Regent's Park, containing twenty-five beds. Here he remained for twenty-five years, and on his retirement was elected a member of the Committee. His last year of office was signalised by the opening of a new building for the hospital, with thirty-eight beds, in Maida Vale, which, including equipment and fittings, cost £29,000. It is noteworthy that he succeeded in securing the incorporation of this hospital before the new building was commenced. Mr. Howgrave Graham was a member of the Committee of Hospital Secretaries appointed to examine into the Uniform System of Accounts, which was followed in 1906 by the appointment of a small committee, including the representatives of the three great funds. Mr. J. G. Griffiths, C.V.O., represented the King's Fund, Mr. R. A. Owthwaite the Hospital Sunday Fund, and the late Mr. W. G. Bunn the Hospital Saturday Fund. After ten meetings and an excellent report the Uniform System of Accounts was enforced by the three great funds, and came into general use throughout the country. For nearly thirty-six years Mr. Howgrave Graham also served as secretary to the Institute of Patent Agents under the Patents Act of 1888, which Institute received a Royal Charter by incorporation in 1891. In his early life he was articled for three years to the late Sir Robert Rawlinson, for many years a trusted and eminent sanitarian and water-supply engineer, who presided over many Commissions and did splendid work in connection with the Lancashire distress arising out of the cotton famine, and in multitudes of ways rendered eminent service at the Local Government Board, whilst he was a leading spirit of the Royal Sanitary Institute. Mr. Howgrave Graham's death occurred, whilst on a short holiday, on the 10th instant, in his eightieth year. He was a very careful, industrious, trustworthy, and devoted worker. He had a multitude of acquaintances and friends, and it is pleasant to think of him resting, as he does, in the beautiful churchyard of Sevenoaks Parish Church, where his wife was laid some years ago. He leaves two sons on active service, one in South Africa, and one engaged on Admiralty work. Mr. Howgrave Graham was well known and respected in the hospital world for many years, and their sympathies will be extended in full measure to all members of his family.

### HOSPITAL PATIENTS FORM A JURY: A PRECEDENT CREATED.

Mr. E. R. Turton, M.P., chairman of the North Riding Quarter Sessions, who is already known for having abolished the practice of calling the grand jury when there were no cases to be heard, on October 17 declared that he had taken the responsibility to issue instructions that no jurymen were to attend. His reason was that, in these times, it was, in his view, a mistake for the Court to summon a large body of gentlemen to attend on the chance of being required. Since he had failed to bring the Home Office to his opinion, he had obtained from the Attorney-General a warrant of *tales*, and, if a prisoner pleaded "Not guilty," he would fill the jury-box with twelve of the gentlemen present in court. On the day in question, the majority of the public was a body of wounded soldiers from the York County Hospital. Mr. Turton, on these being empanelled, said that he could not conceive a better tribunal. After the jury had acquitted a woman charged with attempted suicide, Mr. Turton said, "A very proper verdict, gentlemen." The above is said to be the first instance of such an empanelment. Trial by jury is a system which so far has survived its critics. Will the time come when government by jury will replace the system of government by the Front Benches, disguised as the Party system, in our unhappy land?

### THE BLINDED SOLDIER IN FRANCE.

From the powerful pen of M. Eugène Brieux, of the Académie Française, comes a monograph on the blinded soldiers of France and what is being done for them. This has been translated into English, and is issued by the American Red Cross Institute for the Blind. The first thing to bring home to the blind man is that he can still be happy, chiefly through attaining self-confidence and the desire for independence. It must be urged that they will soon be able to get along without help from others' eyes, and that the law of compensation works within their being, developing other senses to make up for the one that has been lost. This is the theme underlying M. Brieux's pamphlet, which, however, is far from being entirely theoretical. It deals on the practical side with possibilities of occupations, and, having in view the love of the Frenchman for *la terre*, considers especially farming as a pursuit for the blinded soldier. One such, in a letter full of bravery not less than the bravery of the battlefield, admits that he once said: "It is easy for M. Brieux to talk; I should like to know what a man can do that cannot see." Later, having been trained by himself and others in self-confidence, he admits that he could never have believed he could be as contented as he is now, feeding his beasts and digging his beets and his potatoes. The blind themselves pitch the note for their sympathisers. Hope and confidence they seek, but no pity. Some French blind schools display on their walls the warning: "To pity is not to console."

### HOSPITAL SECRETARY APPOINTED GUARDIAN.

The military duties of Mr. Richard Allen, the secretary of the Royal Hospital, Richmond, have permitted a return to the London district after a year's service in France. At a recent meeting of the Richmond Board of Guardians it was resolved, in accordance with the wish of the Government, to fill the existing vacancies without an election. For Richmond North Sheen Ward the election of Mr. Richard Allen, now holding the position of staff-sergeant in the Army, was moved. His proposer referred to Mr. Allen's fine service in France, to his keen interest in work among the poor, and to his knowledge of the work which the Board was trying to do. Although co-option did not appeal to some of the Guardians, the motion was carried by a substantial majority. The whole Poor-Law system at present may be in process of change, but in respect of Boards of Guardians, or whatever local bodies may take their places, the training of a hospital secretary would obviously be useful for subsequent occupation in municipal work.

### THE WINTER AND SIR CYRIL COBB.

Sir Cyril Cobb, Chairman of the London County Council Education Committee, has replied to the complaint of the Camberwell Borough Council that the authorities delayed to provide for the children displaced from the schools which have been taken over as military hospitals. The action taken by the Council was reported in the columns of The Hospital so long ago as July 13. It has taken over two months to receive the reply from Sir Cyril Cobb. In his letter to the Council he states that some of the children have been accommodated at neighbouring schools, but for those children formerly attending two schools, who have been receiving their tuition in the open air, it has now been decided to hire temporary premises, and when the necessary alterations have been carried out it will be possible to permit the children to receive full-time tuition. It is little wonder that the Council has told Sir Cyril Cobb and his committee how very disappointed it is at the latter's delay. In fact the delay is still going on, and it is unlikely that the winter will wait Sir Cyril Cobb's convenience before further complicating the matter.

### THE AMERICAN ST. DUNSTAN'S.

The pensions and military authorities of the United States have not been too proud to take advantage of the experience of St. Dunstan's, that most popular of our war charities, in respect of the training of the blinded sailor or soldier. The American St. Dunstan's is the Red Cross Institute for the Blind, Guilford, Baltimore, Maryland, and is the connecting link between the military and civil life of the men. The institute has also created an Industrial Survey Commission, which is actively engaged in collecting information relative to the employment of the blind in factories. In addition, a women's auxiliary has been organised, by means of which volunteer service is being utilised. The first efforts of the ladies have been in connection with the school and social life of the blind men at Evergreen. The present wages of the average blind man in the States are deplorably low, and a leaf must be taken from the book of St. Dunstan's in discovering new fields to explore and new occupations to develop in order to help the blind to become self-supporting wage-earners. Blind sailors and soldiers are beginning to arrive at Evergreen, and, though it is hoped that the work will not be so extensive as in this country, no one will dissent from the decision of the authorities that, however few the cases are, every possible thing that can be done for them must be done.

### THE MEASURE AND THE MILK-JUG.

The 1917 report of Dr. Howarth, Medical Officer of Health for the City of London, contains evidence of a heavy proportion of adulterated samples of milk and butter. This is attributed to war-time conditions, since the supply of these articles is far below the demand for them. A quarter of the milk samples also contained dirt in recognisable amounts. The proportion showing tubercle bacilli present was, fortunately, comparatively very low.

Complaint is also made of the frequency of short measure. The Government stamp is placed only on metal receptacles, whereas jugs of ware are commonly used to fetch milk. It is recommended that jugs should be used which hold as nearly as possible the exact amount required to be purchased. For the sake of increased cleanliness, the old-fashioned ventilated churn is disappearing and "improved" types are being substituted.

### THE STARTING-POINT OF AFTER-CARE.

Too often the activities of "after-care committees" whittle away to nothing at all, at any rate little useful results. The Herefordshire Insurance Committee has referred to its Sanatorium Benefits Sub-Committee a motion to extend existing arrangements for the after-care of tuberculous persons throughout the county by the formation of district Care Committees to act under the Sanatorium Benefits Sub-Committee; also to approach, if advisable, the Insurance Commissioners as to the possibility of paying sickness benefit to tuberculous insured persons engaged in light work during their gradual return to full working capacity. These motions were introduced by Dr. Gold, who, however, excluded discharged soldiers from their purview, since this class were to be jointly considered by the County Council and the Insurance Committee. Broadly, however, sanatorium patients after discharge needed material help; a proposition with which few will be found to disagree. However, as we have said more than once, just as charity starts at home, so after-care should start at the sanatorium, with the employment of ex-patients.

### POLITESSE A LA POOR LAW.

Boards of Guardians throughout the country have received from the Association of Poor-Law Unions in England and Wales a circular on the subject of a suggested general scheme for the administration of "public assistance." Amongst the recommendations is found a suggestion that certain familiar terms used in Poor-Law work should be clothed in different and more polite language. A pauper, it is urged, might conveniently be called an assisted person, and in this impending Poor-Law millennium relief will be called public assistance and a relieving officer a public assistance inspector. We agree that a workhouse is "an institution," but is that term sufficiently expressive? The change from asylum to mental hospital, if illogical, has something to recommend it, but by what reasoning should a pauper lunatic be called a "local patient"? These ideas have already been referred to in our columns and the opinion expressed that if there is opprobrium attached to-day to the word "pauper," it is sure that the same feeling will be felt in connection with "assisted person" in ten years' time. Already as a term of contempt "insured person" has no rival. But to be an assisted person as well will be found too much. But why a person who has worked and paid rates for many years should feel shame in using institutions he has helped to maintain, whatever he may be called, is a mystery Mr. Pecksniff alone can resolve. But Mr. Pecksniff was an Englishman of the bull-dog breed, and was rarely a bad guide to the feelings of his fellow-countrymen.

### EGGS AND BACON FOR INFIRMARY STAFFS.

The great increase in the price of eggs has led the Southwark Board of Guardians, with the consent of the steward of their infirmary and the chief officer of their Newington institution, now used by the aged and infirm, to alter the ration scale for the staffs. The reduction in the consumption of meat in February from 2½ lb. to 1¼ lb. a week, including bone, was to be replaced by the additional increase of six eggs when obtainable, the cost of these extra eggs not to be in excess of the meat. In consequence of the present high price of eggs they are to be discontinued for the present, and a pound of bacon weekly is to be substituted. This is a departure from the Local Government Board's rationing scheme of last April, but since then bacon has been made available without coupons, and is not included in the meat rations for the general public. Moreover, there is a fairly good supply of bacon. Those in charge of infirmaries and hospitals will have to alter the dietary of their staffs according to the prevailing conditions of the market, and consume whatever is to be had at the time.

### BELGRAVE HOSPITAL FOR CHILDREN.

At a Quarterly Court of Governors of the Belgrave Hospital for Children, Clapham Road, held last week under the presidency of Mr. Thomas F. Ryder, M.V.O. (deputy chairman), it was reported that, although the institution started the present year free from debt, it owed, at the present moment, over £1,550. During the quarter ended September 30, 94 medical and 62 surgical in-patients had been cared for, while 905 medical and 922 surgical out-patients, whose attendances totalled 9,121, had also received attention. The hospital had also attended to over 600 casualties. The Earl of Plymouth (chairman of the institution) was unable to be present owing to serious illness.

### INFANTILE MORTALITY AND THE USE OF COTS.

The teaching of Dr. F. J. Waldo, Coroner for the City of London and the Borough of Southwark, who has persistently advised mothers to use cots, seems after many years to be bearing fruit. Last year in his district the number of deaths of infants accidentally suffocated whilst sleeping with their parents was only 550, as compared with 1,100 in 1912, and 1,090 in 1914. Please note that Dr. Waldo considered that the liquor restrictions, to which the Registrar-General attributed the decline, were a minor factor. The chief cause, in his opinion, was the greater use of cots and cradles by the poor, the more general ordering of *post-mortem* examinations in such cases, and, through the absence from home of many husbands on active service, the lessening of the number of persons sleeping in the same bed. He was of opinion that the slightly larger number of these cases occurring between Saturday and Monday, and on public holidays, was due to the harder work and sounder sleep of the parents, and not to intoxication. This is refreshingly definite. Who is not convinced?

### COMMANDEERED MILK FOR INFANTS.

According to a report of the Willesden Council the Food Committee, owing to the restricted supply of sweetened condensed milk, decided to enforce the Food Control Committee's Requisitioning Order, 1918, and thereby to ensure during the winter a regular supply of condensed milk for infants and for invalids. In accordance with the powers conferred by the Order, certain traders in each ward have been directed to hold at the disposal of the Food Office their entire consignments of sweetened condensed milk, and to dispose of them only upon production of a voucher signed by the executive officer.

### THE PLUMSTEAD INFANTS' HOSPITAL.

With the help of the American Red Cross, which has formed a new centre at the Central Hall, Plumstead, the Woolwich Borough Council has organised a Maternity and Child Welfare Centre and observation ward at Perran Lodge, Eglington Road, Plumstead. On the occasion of the opening Dr. Truby King gave an address to the assembled mothers.

### THE SALARIES OF HEALTH VISITORS.

The twenty-six health visitors in the service of the Willesden Urban District Council applied for an increase of £20 per annum. The matter has been before the Health Committee, which decided to accede to the application by fixing the maximum scale salary, on the permanent staff, at £20 a year more.

### FOR COTTAGE HOSPITAL MATRONS.

A contemporary quotes, with a shrug of disapproval, the boast of "a society lady" to the effect that a cottage hospital did not cost her and her daughter so much as a season in London. Whatever may be thought of the remark itself, it appears to have been applied to a house used as a convalescent home for soldiers. These, alas! have a varied standard, which tends to depend upon the character of those who "run" them. Cottage hospital matrons will hardly feel complimented on the comparison of their institutions, which have a tradition and a standard, to those which, however good in some cases, have none of the first and an uncertain degree of the second.

### "THE HAPPY HOSPITAL."

Uniform with the volume possessing this agreeable title, as we learn from the *Gazette* of the 3rd London General Hospital, Wandsworth, Messrs. Simpkin, Marshall have published a cheap edition of the "Observations of an Orderly," by Ward Muir. The reprint, which costs now 1s. 6d. (it was published originally at half-a-crown), will be welcome, and those who read it on publication and learnt to take a friendly interest in its author will be intrigued to know that he is now engaged on propaganda in the *Zona di Guerra, Italia*. The *Gazette* itself, however, has merrily survived the absence of that excellent contributor. Indeed, the chief praise earned by the original members of the staff has been to create a tradition to the making of which each contributed, but the embodiment of which was not confined to two or three. Miss Nightingale continues to cast the sensations excited in her mind by the war into verse, fable, and pleasant prose. No one else seems to have her peculiar flavour; her style reminds us of the scent of an English russet, one of the few which with rough skin and red side survived the frosts and winds of March. The cartoon, for so it is, in the October number by Private H. M. Hemsley, entitled "The Incorrigibles," is tragi-comic in its directness, and we are glad that the figure of the nurse is so placed as to disguise half the sting of the incident. An armless man is a sorry sight, but a legless man a cruel one. Private Hemsley has an eye for a hard fact, and his sentiment without tears has a tonic value. Donovan, another incorrigible of a less subtle kind, continues to inspire Lance-Corporal J. H. Dowd. Artistically, Sergeant G. J. Coates' "A Hand at Cards" is the best thing in the current number.

### THIS WEEK'S DRUG MARKET.

The announcement that dealings in quinine will shortly be regulated by the Army Council under the Defence of the Realm Regulations, and that a schedule of fixed prices will be issued to apply to all sales made after the date of the Order, is the most important event of the week so far as the drug market is concerned. It is as yet too early to estimate what is likely to be the full effect of this announcement in the quinine market during the period that will elapse between now and the publication of the promised Order, but buyers who can carry on until the Order comes into force will hardly be disposed to make purchases at the present time. The demand for quinine has been heavy as a result of the epidemic of influenza, and on the other hand supplies of quinine available to the public through the ordinary channels of trade have been comparatively very small. The consequence is that prices have recently advanced substantially, and those holders who had bought stocks only a short time ago are now making handsome profits. It is useless to forecast what the fixed prices will be, but we commend *Punch's* advice to those who are about to buy quinine. Imported saccharin still has a downward tendency in price. The prices of menthol and Japanese peppermint oil are fairly well maintained, and eucalyptus oil still has an upward tendency in value. Phenazone is rather dearer.

### TO OUR READERS.

**Contributions are specially invited from any of our readers to these columns. They should deal with topical subjects and news. They must be authenticated for the information of the Editor only. The minimum payment if published is 5s. There is no hard-and-fast rule as to space, but notes of about twenty lines in length are preferred.**

